# Glycated lysine-141 in haptoglobin improves the diagnostic accuracy for type 2 diabetes mellitus in combination with glycated hemoglobin HbA_1c_ and fasting plasma glucose

**DOI:** 10.1186/s12014-017-9145-1

**Published:** 2017-03-28

**Authors:** Sandro Spiller, Yichao Li, Matthias Blüher, Lonnie Welch, Ralf Hoffmann

**Affiliations:** 1 Institute of Bioanalytical Chemistry, Faculty of Chemistry and Mineralogy, Universität Leipzig, Leipzig, Germany; 2 Center for Biotechnology and Biomedicine (BBZ), Universität Leipzig, Deutscher Platz 5, 04103 Leipzig, Germany; 3 School of Electrical Engineering and Computer Science, Ohio University, Athens, OH USA; 4 Department for Internal Medicine, Clinic for Endocrinology and Nephrology, University Hospital Leipzig, Universität Leipzig, Leipzig, Germany

**Keywords:** Biomarker, Fasting plasma glucose (FPG), Glycation sites, HbA_1c_, Multiple reaction monitoring (MRM), Plasma proteins, Protein glycation, Type 2 diabetes mellitus

## Abstract

**Background:**

Recent epidemiological studies indicate that only 30–50% of undiagnosed type 2 diabetes mellitus (T2DM) patients are identified using glycated hemoglobin (HbA_1c_) and elevated fasting plasma glucose (FPG) levels. Thus, novel biomarkers for early diagnosis and prognosis are urgently needed for providing early and personalized treatment.

**Methods:**

Here, we studied the glycation degrees of 27 glycation sites representing nine plasma proteins in 48 newly diagnosed male T2DM patients and 48 non-diabetic men matched for age (range 35–65 years). Samples were digested with trypsin and enriched for glycated peptides using boronic acid affinity chromatography. Quantification relied on mass spectrometry (multiple reaction monitoring) using isotope-labelled peptides as internal standard.

**Results:**

The combination of glycated lysine-141 of haptoglobin (HP K141) and HbA_1c_ provided a sensitivity of 94%, a specificity of 98%, and an accuracy of 96% to identify T2DM. A set of 15 features considering three glycation sites in human serum albumin, HP K141, and 11 routine laboratory measures of T2DM, metabolic syndrome, obesity, inflammation, and insulin resistance provided a sensitivity of 98%, a specificity of 100%, and an accuracy of 99% for newly diagnosed T2DM patients.

**Conclusions:**

Our studies demonstrated the great potential of glycation sites in plasma proteins providing an additional diagnostic tool for T2DM and elucidating that the combination of these sites with HbA_1c_ and FPG could improve the diagnosis of T2DM.

**Electronic supplementary material:**

The online version of this article (doi:10.1186/s12014-017-9145-1) contains supplementary material, which is available to authorized users.

## Background

Diabetes mellitus (DM) refers to a group of metabolic disorders characterized by elevated blood glucose concentrations. The majority (90–95%) of DM patients have type 2 DM (T2DM), which is characterized by peripheral insulin resistance and an inability of pancreatic beta cells to compensate for that by increasing insulin secretion [1]. Current diagnostic criteria of the World Health Organization (WHO) for the diagnosis of T2DM include glycated hemoglobin (HbA_1c_) levels (≥6.5% (48 mmol/mol)), elevated fasting plasma glucose (FPG) concentrations (FPG ≥ 7.0 mmol/L) and/or plasma glucose concentrations 2 h after a 75 g oral glucose load (≥11.1 mmol/L) in the context of a standardized oral glucose tolerance test (OGTT) [2, 3]. The American Diabetes Association (ADA) defines a HbA_1c_ level of ≥6.5% or a fasting plasma glucose (FPG) level of ≥126 mg/dL (≥7 mmol/L) or 2-h plasma glucose level of ≥200 mg/dL (≥11.1 mmol/L) during a 75-g oral glucose tolerance test (OGTT), or a random plasma glucose of ≥200 mg/dL (≥11.1 mmol/L) as equally valid diagnostic criteria for type 2 diabetes [1]. However, epidemiological studies revealed that compared to OGTT glucose values the use of either HbA_1c_ or FPG only identifies ~30–50% of previously undiagnosed T2DM patients [4–8]. Moreover, categories of increased risk for diabetes (prediabetes) include a HbA_1c_ level of 5.7–6.4% or a FPG level between 100 and 125 mg/dL (5.6–6.9 mmol/L) or 2-h plasma glucose level between 140 and 199 mg/dL (7.8–11.0 mmol/L) in the OGTT [1]. For the distinction of patients with prediabetes, HbA_1c_ values have also been shown to be less sensitive compared to FPG and 2 h OGTT glucose [7]. HbA_1c_ also plays a critical role in the management of diabetic patients, as it reflects the average plasma glucose level for the preceding 3-month period and correlates well with micro- and macrovascular complications [4–6]. FPG is the preferred diagnostic parameter, because it is simple, inexpensive, relatively risk-free, and correlates with diabetic complications like retinopathy [7]. However, even using longitudinally measured HbA_1c_ levels, the prediction or retrospective attribution of individual T2DM associated complications or mortality are not possible with this parameter of chronic hyperglycemia [8–12]. Therefore, there is an unmet need to better predict the individual chronic hyperglycemia-related diabetes outcomes.

Glycated proteins, such as hemoglobin in erythrocytes and serum albumin as major plasma protein, are recognized as markers of hyperglycemia due to the high sensitivity towards even slightly elevated plasma glucose levels [13, 14]. However, only methods determining global protein glycation degrees have been established, whereas recent evidence suggests specific glycation sites in plasma proteins as potential biomarkers for early diagnoses of DM [14–17]. In contrast to the long half-life time of hemoglobin in erythrocytes, plasma proteins vary in half-life times from hours to several weeks, which might allow selecting a small set of protein glycation sites resembling more closely short to medium term fluctuations of plasma glucose levels.

Here we quantified 27 glycation sites in nine plasma proteins after tryptic digestion by mass spectrometry and evaluated their diagnostic value alone and in combination with current WHO criteria of HbA_1c_ and FPG levels. Selection of glycation sites relied on a list of 52 candidates we previously identified and studied [17], by judging each for the biomarker potential and ease of peptide synthesis. A single-blind study using plasma samples from 48 newly diagnosed T2DM patients and 48 non-diabetic individuals clearly indicated different glycation degrees characteristic of each glycation site with glycated Lys141 of haptoglobin providing the best sensitivities (~94 and ~78%) and specificity (~98%) when combined with HbA_1c_ and FPG levels, respectively.

## Methods

### Blood samples

Blood samples were obtained from 48 newly diagnosed male T2DM patients and 48 non-diabetic men matched for age (range 35–65 years) in the context of a study of parameters of insulin resistance (Additional file 1: Table S1). Anthropometric and laboratory chemistry parameters were measured or calculated as previously described [18, 19]. The study was approved by the Ethics Committee of Universität Leipzig (approval no: 159-12-21052012), and performed in accordance to the declaration of Helsinki. All subjects gave written informed consent before taking part in this study. In our study, we defined individuals with T2DM according to the criteria of the American Diabetes Association [1]:

- A HbA_1c_ level of 6.5% or higher, or
- A fasting plasma glucose (FPG) level of 126 mg/dL (7 mmol/L) or higher (fasting was defined as no caloric intake for at least 8 h), or
- A 2-h plasma glucose level of 200 mg/dL (11.1 mmol/L) or higher during a 75-g oral glucose tolerance test (OGTT), or
- A random plasma glucose of 200 mg/dL (11.1 mmol/L) or higher in a patient with classic symptoms of hyperglycemia (i.e., polyuria, polydipsia, polyphagia, weight loss) or hyperglycemic crisis

The group of T2DM patients has been further categorized into those with HbA_1c_ levels patients below (n = 23) and equal or higher than 6.5% (48 mmol/mol) (n = 25). For those patients with HbA_1c_ < 6.5% (48 mmol/mol), the diagnosis of T2DM has been established on the basis of repeated measurements of fasting plasma glucose (>7.0 mmol/L) or 2 h oral glucose tolerance test glucose concentrations >11.1 mmol/L [1]. Since patients were newly diagnosed, none of the individuals in the T2DM receive anti-hyperglycemic medication. All men had a body mass index (BMI) larger than 25.0 kg/m^2^ and were grouped in overweight (BMI 25–30 kg/m^2^; 15 without and 10 with T2DM) and obese individuals (BMI ≥ 30 kg/m^2^). EDTA blood samples were collected between 8 a.m. and 9 a.m. after a 12-h fasting period, centrifuged (500×*g*, 5 min) and analyzed within 1 h after blood drawing for routine laboratory parameters or stored at −80 °C after the cell debris was removed by filtration (Rotilabo^®^ syringe filter) for the analyses of the glycation sites. Plasma insulin and proinsulin were measured with an enzyme immunometric assay for the IMMULITE automated analyzer (Diagnostic Products Corporation, Los Angeles, CA, USA). Serum high-sensitive CRP (C-reactive protein) was measured by immunonephelometry (Dade-Behring, Milan, Italy). HbA_1c_, plasma glucose, serum total- high-density lipoprotein (HDL)-, low-density lipoprotein (LDL)-cholesterol, triglycerides, and free fatty acids were measured as previously described [18].

### Peptide quantification

Glycation sites previously identified in plasma samples of patients with diabetes [17] were quantified by electrospray ionization mass spectrometry (ESI–MS) on a QTRAP 4000 (AB Sciex, Darmstadt, Germany) coupled on-line to reversed-phase high-performance liquid chromatography (RP-HPLC) using timed multiple reaction monitoring (MRM) (Table 1 and Additional file 1: Table S2). Briefly, small molecules and peptides were removed from plasma by ultrafiltration (5 kDa cut-off). The concentrated sample was digested with trypsin (37 °C, 18 h, 5% w/w), spiked with a concentration-balanced mixture of ^13^C,^15^N-labelled glycated peptides as internal standard, enriched for glycated peptides by boronic acid affinity chromatography (BAC), and desalted by solid phase extraction (SPE) using optimized protocols [15, 20–22]. The internal standard was added after tryptic digestion to provide the highest accuracy by compensating variations, such as nonspecific degradation, during sample preparation [23], especially as trypsin could cleave some standard peptides at Lys/Arg-Pro-motifs or glycated residues.

**Table 1:** Glycated peptides of different plasma proteins quantified in tryptic digests of type 2 diabetes and control plasma samples

#	Sequence	Protein symbol (Accession number, glycation site)	*t*_*R*_ (min)	Q1 *m/z *(±0.2)	Q3 *m/z* (±0.2)
1	TCVADESAENCD**K**SLHTLFGDK	HSA (P02768; K64)	13.8	887.1	869.1
2	SLHTLFGD**K**LCTVATLR	HSA (P02768, K73)	17.9	698.7	680.7
3	ETYGEMADCCAKQEPER	HSA (P02768, K93)	8.8	746.0	136.1
4	ETYGEMADCCA**K**QEPER	HSA (P02768, K93)	7.9	751.3	136.1
5	AAFTECCQAAD**K**AACLLPK	HSA (P02768, K174)	15.9	763.0	120.0
6	AACLLP**K**LDELRDEGK	HSA (P02768, K181)	16.2	664.0	646.0
7	AEFAEVS**K**LVTDLTK	HSA (P02768, K233)	18.6	907.0	880.0
8	ADLA**K**YICENQDSISSK	HSA (P02768, K262)	13.6	1052.5	1025.5
9	TYETTLE**K**CCAAADPHECYAK	HSA (P02768, K359)	9.9	670.8	237.1
10	VFDEF**K**PLVEEPQNLIK	HSA (P02768, K378)	18.6	1104.1	1077.1
11	**K**VPQVSTPTLVEVSR	HSA (P02768, K414)	14.9	601.3	900.5
12	**K**QTALVELVK	HSA (P02768, K525)	13.1	645.9	603.9
13	EQL**K**AVMDDFAAFVEK	HSA (P02768, K545)	18.7	668.3	120.1
14	**K**LVAASQAALGL	HSA (P02768, K574)	16.3	652.4	501.8
15	VQW**K**VDNALQSGNSQESVTEQDSK	IGKC (P01834, K41)	12.7	947.1	941.1
16	DSTYSLSSTLTLS**K**ADYEK	IGKC (P01834, K75)	16.4	757.7	751.7
17	VYACEVTHQGLSSPVT**K**SFNR	IGKC (P01834, K99)	12.6	848.1	830.1
18	QV**K**DNENVVNEYSSELEK	FGB (P02675, K163)	12.8	762.7	72.1
19	S**K**AIGYLNTGYQR	A2M (P01023, K1003)	10.5	816.9	1255.6
20	ALLAYAFALAGNQD**K**R	A2M (P01023, K1162)	18.5	628.7	86.1
21	**K**CSTSSLLEACTFR	TF (P02787, K683)	13.8	911.4	884.4
22	ADSSPV**K**AGVETTTPSK	IGLC (P01842, K50)	8.7	613.0	70.1
23	A**K**VQPYLDDFQK	APOA1 (P02647, K120)	12.5	807.4	765.4
24	**K**WQEEMELYR	APOA1 (P02647, K131)	10.1	530.6	524.6
25	AVGD**K**LPECEAVCGKPK	HP (P00738, K141)	9.4	674.0	656.0
26	M **K**GLIDEVNQDFTNR	FGA (P02671, K71)	14.9	653.3	894.4
27	SSSYS**K**QFTSSTSYNR	FGA (P02671, K581)	8.7	664.6	658.6

The timed multiple reaction monitoring relied on the retention time of each peptide in RP-HPLC and a specific precursor/fragment ion pair (Q1/Q3 mass range)

*t*_*R*_ retention time, *C,* *M* *, and* ***K*** carbamidomethylated cysteine, methionine sulfoxide, and fructosamine lysine, *HSA* human serum albumin, *IGKC* Ig kappa chain c region, *FGB* fibrinogen beta chain, *A2M* alpha-2-macroglobulin, *TF* serotransferrin, *IGLC* Ig lambda chain C region, *APOA1* apolipoprotein A-I precursor, *HP* haptoglobin, *FGA* fibrinogen alpha chain

Peptides were loaded on a C_18_-column (AdvanceBio Peptide Mapping column, pore size 120 Å, length 150, 2.1 mm internal diameter, 2.7 µm particles, Agilent Technologies, Böblingen, Germany) coupled on-line to ESI–MS. Eluents A and B were water and acetonitrile, respectively, containing both formic acid (0.1%, v/v). Elution was achieved by a linear gradients starting 3 min after sample injection from 3 to 10% eluent B within 1 min, to 20% eluent B within 10 min and to 95% eluent B in 7 min. The flow rate was 0.3 mL/min and the column temperature was set to 60 °C. Quantification relied on timed MRM using specific transitions of each targeted peptide and isotope-labelled internal peptide standards synthesized on solid phase in-house. Quantification was performed by integrating individual peaks in extracted ion chromatograms (XICs) using Analyst 1.6 software (AB Sciex) relative to the coeluting isotope-labelled peptides.

### Statistics and bioinformatics

The samples from individuals with or without T2DM were evaluated by different statistical tests (Kolmogorow-Smirnow, Mann–Whitney, and *t* test) and calculation of Spearman rank correlation coefficients using Prism 6 (GraphPad software; La Jolla, USA). Receiver operating characteristic (ROC) analysis and screening for variable combination relied on the Excel-add-in Multibase 2015 (Numerical Dynamics) and Prism 6 software, respectively.

The 48 diabetic and 48 samples were classified by a decision tree algorithm using HbA_1c_ in combination with each glycated peptide (Additional file 1: Table S6). The same technique was also applied to FPG (Additional file 1: Table S7). The decision tree algorithm was implemented using Scikit-Learn [24]. Accuracies were evaluated using nested tenfold cross validation [25]. To find the best feature set for classification, a support vector machine-recursive feature elimination (SVM-RFE) method [26] was applied on all glycated peptides and clinical parameters, including HbA_1c_, FPG, BMI, etc. Feature normalization and missing value imputation were performed using WEKA toolkit [27]. The support vector machine was implemented using Scikit-Learn [24]. Accuracies and area under the curve (AUC) values were evaluated using nested tenfold cross validation [25]. Hierarchical clustering was performed on 48 diabetic samples using the “hclust” function in R software version 3.2.1 [28]. To find the subclasses in diabetic samples, the expectation–maximization algorithm in Scikit-Learn [24] was applied. The clustering stability score [29] and elbow criterion [30] were used to find the optimal number of subclasses.

## Results

The timed MRM method optimized for quantification of the targeted 27 glycated peptides (Table 1) obtained by tryptic digestion from plasma provided limits of detection (LODs) and quantification (LOQs) in the low to high nanomolar range (Additional file 1: Table S3). Intra- and interday precisions showed typically coefficients of variation (CVs) below 20% (Additional file 1: Table S3). The quantities of all 27 glycated peptides normalized to the total protein content of each plasma sample were significantly higher in T2DM than in the control group (P < 0.05, Additional file 1: Figure S1). However, the normalized quantities of all glycated peptides showed a notable overlap. Interestingly, diabetic groups subdivided by an HbA_1c_ threshold of 6.5% (48 mmol/mol) showed very similar average glycation degrees for all peptides, indicating that the glycation degree of hemoglobin in erythrocytes showed only a weak correlation with the glycation levels of serum proteins providing a rationale that they may have different diagnostic and prognostic values.

Spearman’s correlation coefficients (*r*_*S*_) calculated for each glycated peptide amount and the established diagnostic parameters indicated moderate correlations between a few glycation sites and BMI (−0.54 < *r*_*S*_ < −0.38, P < 0.001), body fat (−0.49 < *r*_*S*_ < −0.36, P < 0.01), and C-peptide levels (−0.45 < *r*_*S*_ < −0.37, P < 0.001) (Additional file 1: Table S4). Correlations between peptide glycation levels and HbA_1c_ were weak (0.11 < *r*_*S*_ < 0.34, 0.001 < P < 0.3) to moderate (0.37 < *r*_*S*_ < 0.46, P < 0.001) and also weak (−0.08 < *r*_*S*_ < 0.30, 0.001 < P < 0.65) for FPG (Additional file 1: Table S4). Besides FPG (*r*_*S*_ = 0.40, P < 0.001) and free fatty acids (FFA, (*r*_*S*_ = 0.57, P < 0.001) HbA_1c_ showed for further diagnostic parameters only weak correlations (−0.35 < *r*_*S*_ < 0.35, 0.001 < P < 0.95). However, FPG showed moderate correlations with fasting plasma insulin (FPI*, r*_*S*_ = 0.40, P < 0.001), proinsulin (*r*_*S*_ = 0.38, P < 0.01), homeostasis model assessment as an index of insulin resistance (HOMA-IR, *r*_*S*_ = 0.56, P < 0.001), and fasting plasma free fatty acids (FFA) (*r*_*S*_ = 0.56, P < 0.001).

A ROC curve analysis for samples from T2DM patients relative to HbA_1c_ (cut-off 6.5% (48 mmol/mol)) and FPG (cut-off 7.0 mmol/L) provided for all glycated peptides maximal sensitivities and specificities of 79 and 88%, respectively, and AUCs larger than 79% for certain cut-off concentrations (Additional file 1: Table S5). In comparison, sensitivities, specificities, and AUCs of HbA_1c_ were 52, 100, and 86%, respectively, as well as 40, 100, and 84% for FPG using the aforementioned cut-offs. A ROC curve analysis determined the best cut-off values maximizing sensitivity and selectivity as 6.0% (42 mmol/mol) for HbA_1c_ (77 and 94%) and 5.69 mmol/L for FPG (75 and 81%).

**Fig. 1:**
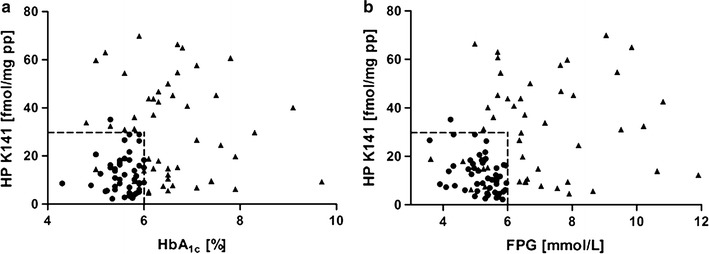
Scatter plots of HbA_1c_ values (**a**) and fasting plasma glucose (FPG) levels (**b**) against peptide levels corresponding to Lys141 of haptoglobin (HP K141). Numerical values of 48 type 2 diabetes patients *(black triangles*) and 48 control persons (*black circles*) are shown. *Dashed lines* illustrate the cut point chosen for each feature

As the diagnostic accuracies of HbA_1c_ (76% for cut-off 6.5% (48 mmol/mol), 86% for cut-off 6.0%), FPG (70% for cut-off 7.00 mmol/L, 78% for cut-off 5.69 mmol/L), and the evaluated glycated peptides (60 to 73%) were insufficient, the data sets were screened by decision tree algorithm for variable combinations of HbA_1c_ or FPG with any glycated peptide for optimal sensitivity, specificity, and accuracy using different cut-off points. HbA_1c_ in combination with glycated peptides yielded mostly sensitivities from 75 to 81%, specificities from 96 to 100%, and accuracies from 87 to 90% (Additional file 1: Table S6). Most interestingly, analyses revealed a haptoglobin peptide glycated at Lys141 (HP K141; sequence No. 25) which provided in combination with HbA_1c_ a sensitivity of 94%, a specificity of 98%, and an accuracy of 96% for cut-off points 6.0% (42 mmol/mol, HbA_1c_) and 30 fmol/mg (HP K141) (Fig. 1a). When HP K141 was combined with FPG (6.0 mmol/L), sensitivity to detect T2DM was 78%, specificity 98%, and accuracy 88% (Fig. 1b), whereas the other glycated peptides typically provided slightly lower accuracies (Additional file 1: Table S7). Since the decision tree algorithm did not deliver all cut-off values for all combinations, manual confirmation was carried out. Combining manually HbA_1c_ or FPG with glycated peptides provided slightly enhanced diagnostic accuracies for all combinations and could confirm all cut points provided by the algorithm with only small variations (Additional file 1: Tables S8 and S9). Noteworthy, the combination of HP K141 and FPG (6.0 mmol/L) was improved to a sensitivity of 83%, a specificity of 98%, and an accuracy of 91%.

**Fig. 2:**
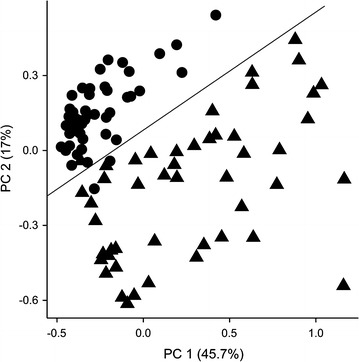
Principle component plot shows the clear separation between T2DM patients and controls using the 15 diagnostic parameters: HbA_1c_, fasting plasma glucose (FPG), free fatty acids (FAA), triglyceride levels, leukocytes levels, C-reactive protein levels, HOMA-IR, age, waist, waist-to-hip ratio, diastolic blood pressure, and glycated peptides 4, 8, 11, and 25 (Table 1). Values for 48 type 2 diabetes patients (*black triangles*) and 48 control persons (*black circles*) are shown. *PC1* first principle component, *PC2* second principle component

The SVM-RFE method was applied to find a set of diagnostic parameters and peptide amounts for maximizing the classification of T2DM patients and controls. It revealed a set of 15 features providing a sensitivity of 98%, a specificity of 100%, and an accuracy of 99% (Fig. 2).

**Fig. 3:**
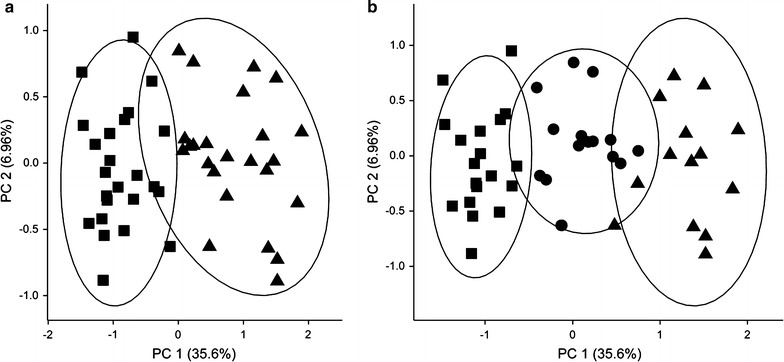
Principle component plot of the clustering of 48 T2DM patients into two (**a**) or three (**b**) clusters based on all available patient information, i.e. diagnostic parameters and glycated peptide levels. Missing values were imputed using Weka. The ellipses are drawn at 95% confidence interval using the *level* parameter in ggplot. *PC1* first principle component, *PC2* second principle component

The cluster analysis of the 48 T2DM plasma samples was applied to an expectation–maximation (EM) algorithm considering all glycated peptides and clinical parameters available (27 glycated peptides + 38 clinical parameters). The result showed two or maximal three clusters (Fig. 3). Glycated peptide levels were distinct among different groups, however, clinical parameters, including HbA_1c_ and FPG, were similar. This suggests that glycated peptides can be more useful to detect subtle differences among diabetic patients. A principle component plot is used to visualize the distribution of clusters (Fig. 3). A cluster stability test considering the elbow criterion identified three clusters as optimal number. This was further confirmed by hierarchical clustering that also suggested three groups of T2DM patients (Additional file 1: Figure S2).

## Discussion

Protein glycation as diagnostic criterion has been addressed in many studies for decades [13–17]. Surprisingly, only glycation of intracellular hemoglobin is currently considered as biomarker to monitor patients assuming that HbA_1c_ levels closely reflect average glucose blood levels over the previous 3 months. Plasma proteins have been mostly analyzed for their global glycation degrees [especially human serum albumin (HSA)], which can be attributed to the lack of specific immunoassays, i.e. antibodies recognizing individual glycation sites. As an example, measurement of fructosamine determines the fraction of total serum proteins (mainly serum albumin) that have undergone glycation. Because albumin has a half-life of approximately 3 weeks, the plasma fructosamine concentration reflects relatively recent changes in blood glucose and is therefore not commonly used to monitor diabetes treatment [31].

Additionally, mass spectrometry has been typically applied for mapping glycation sites in serum proteins, whereas quantitative studies on distinct glycation sites in larger patient cohorts are missing. Besides the analytical challenges, this is probably attributed to the general assumption that glycation as a non-enzymatic reaction depends only on the glucose concentration and thus the glycation levels of individual sites will change at similar degrees and thus can be judged from global glycation degrees. Here, we could show that the glycation levels of 27 sites in nine plasma proteins provide significantly different sensitivities, specificities and accuracies for classifying T2DM patients and controls. Most importantly, these glycation degrees correlated only slightly to HbA_1c_ and other clinical parameters like FPG. Additionally, sensitivities of the newly identified glycation sites were mostly better than for HbA_1c_, while specificities were lower demonstrating that T2DM diagnosis might benefit from combining currently applied clinical parameters with plasma protein glycation sites analyzed in the context of this study. Despite studying 27 glycation sites, it was our aim to identify the diagnostically most relevant modification to keep the number of biomarkers and thus the costs of the envisaged diagnostic tools low. Different statistical analyses identified glycation of Lys141 in haptoglobin as the best parameter in combination with HbA_1c_ and FPG. The obtained sensitivity (94%), specificity (98%), and accuracy (96%) of HP K141 in combination with HbA_1c_ exceeded the corresponding values of HbA_1c_ significantly. This suggests that in addition to HbA_1c_ measurements, glycation of Lys141 in haptoglobin could be used as a diagnostic tool in patients with T2DM and those with a high risk to develop T2DM (Fig. 1a). Further studies are necessary to test whether combination of these two parameters of chronic hyperglycemia may better predict individual risk for the development of diabetes complications. However, an earlier diagnosis of T2DM may be important for individual patients’ outcomes, as it has been demonstrated that a “bad glycemic memory” may contribute to a higher risk of diabetes complications even after periods of well-controlled hyperglycemia [32]. The negative correlation between HP K141 and both BMI and C-peptide appears interesting, as it might indicate higher glycation levels in T2DM patients are caused by both diabetes-specific, but also obesity-associated metabolic alterations, which subsequently may cause chronic hyperglycemia. Negative correlations between glycation levels and BMI suggest that adipose tissue in patients with obesity may have a higher capacity to take up excess glucose before it contributes to glycation of circulating proteins. However, this hypothesis has to be formally proven by glucose distribution studies in lean versus obese animal models under hyperglycemic conditions.

Although our initial intention was to identify a single biomarker, statistical evaluation of the data revealed a promising features selection matrix using three glycation sites in HSA (K93, K262, and K414) besides HP K141 representing short-to-medium term glucose fluctuations in combination with twelve routine parameters typically used to characterize T2DM (FPG, HbA_1c_, fasting insulin), metabolic syndrome (triglycerides and blood pressure), obesity (BMI, waist circumference, waist-to-hip ratio), inflammation (leukocytes, C-reactive protein), and insulin resistance (HOMA-IR), and age [33]. This feature set provided an extremely high accuracy of 99% for the sample cohort providing the best diagnostic value that has been reported to the best of our knowledge. For example, a feature set reported in 2009 relied on 160 individuals (Danish Inter99 prospective study), who progressed from initially non-diabetic to diabetes during the following five years. The predictive values of 58 biomarkers (selected from presumed diabetes-associated pathways) together with six routine clinical parameters were evaluated by statistical learning methods [34]. The best features, i.e. six biomarkers (adiponectin, C-reactive protein, ferritin, interleukin-2 receptor A, glucose, and insulin), were used in a PreDx diabetes risk score (DRS) model providing an AUC of 0.76 that increased to 0.78 when family history, age, BMI, and waist circumference were added. A later report indicated an AUC of 0.838 [35]. The PreDx DRS model outperformed single variables like FPG (~0.73) and HbA_1c_ (~0.66) significantly. In this respect the 15 feature set selected here considering only four new biomarkers besides routine laboratory measures, provides a better diagnostic accuracy with on the AUC of 0.99375 (accuracy 99%).

Another intriguing result of statistical evaluation and hierarchical clustering was the separation of diabetes patients in three subgroups based on all patient information including all 27 glycation sites (Fig. 3; Additional file 1: Figure S2). At this stage, the clinical relevance remains open, but it is compelling to speculate that the subgroups might represent distinct subgroups of T2DM. This hypothesis is supported by a recent report that three T2DM subgroups have been dissected through topological analysis of patient similarity [36]. In this analysis, one T2DM subtype was characterized by diabetic nephropathy and retinopathy, another showed enriched risk for malignancies and cardiovascular diseases, whereas the third subtype correlated most strongly with cardiovascular diseases, neurological diseases, allergies, and specific infections. Based on this data, future longitudinal studies, which assess the risk of these disease entities in addition to the glycation sites described in our study should be performed to test the hypothesis that these three subtypes could be distinguished by protein glycation markers.

These subgroups may also respond differently to multifactorial T2DM treatment strategies or may predict the progression of the disease itself and the individual risk for developing diabetes complications. Noteworthy, our study has some limitations with regard to the small size of our discovery cohort and the limited transferability into clinical practice. Although we only included newly diagnosed T2DM patients at the earliest possible time point and without antidiabetic treatment, we cannot relate the time point of diagnosis to a defined metabolic state. In addition, we could not exclude individual differences in the duration and severity of the prediabetic phase including chronic effects of intermittent glucose and lipid toxicity. Moreover, the suggestion that glycated Lys141 in haptoglobin may improve diagnostic accuracy for T2DM needs to be tested in prospective studies and cohorts representing the common population.

In conclusion, the 27 glycation sites quantified here provided sensitivities up to 79% and specificities of up to 88% to distinguish T2DM samples relative to age- and BMI-matched control samples using specific cut-offs for each glycation site. The cut-off values of HbA_1c_ (6.5% (48 mmol/mol)) and FPG (7.0 mmol/L) recommended by the WHO showed better specificities of 100%, but lower sensitivities of only 52 and 40%, respectively. Interestingly, lowering the cut points to 6.0% (42 mmol/mol) and 5.69 mmol/L improved the sensitivity significantly to 77 and 75%, respectively, while the selectivity was reduced to 94 and 81%, respectively. This is in good agreement with recent reports suggesting that lower HbA_1c_ and FPG cut points will dramatically increase diagnostic sensitivity [37–41]. Remarkably, plasma proteins appeared to follow other glycation kinetics than hemoglobin, which might be related to their different environment (i.e. blood versus erythrocytes). Thus, glycation sites in plasma proteins may provide an additional diagnostic tool, as confirmed here by combining the glycation levels HbA_1c_ and HP K141 providing high sensitivity (94%) and specificity (98%). The advantage of combining these two markers are the different half-life times of the corresponding proteins, i.e. 3–4 months for intracellular hemoglobin and two to four days for haptoglobin (*t*_1/2_ = 2–4) [42] being sensitive to long- and short-term fluctuations of blood glucose concentrations. Furthermore, the combination of 15 features consisting of established clinical parameters and several glycated plasma proteins allowed dividing the newly diagnosed patients with T2DM into three different subgroups supporting the hypothesis that heterogeneity of T2DM phenotypes maybe due differentially affected pathways including significant differences in protein glycation.

## Additional file

**Additional file 1. Table S1**. Characterization of type 2 diabetes patients and matched non-diabetic persons enrolled in this study. **Table S2**. Parameters and settings used for the RP-HPLC-ESI-QqLIT-MS operating in scheduled multiple reaction monitoring (MRM) mode. **Table S3**. Precision, sensitivity and linearity parameters for glycated peptides. **Table S4**. Spearman rank correlation coefficients (rS) and corresponding P values (P) of the statistical relation between glycated peptides and several diagnostic parameters. **Table S5**. Receiver operating characteristic (ROC) parameters for peptide levels of all 27 glycated peptides quantified in tryptic digests of plasma samples obtained from 48 type 2 diabetes patients and 48 controls. For comparison, ROC parameters of HbA1C and fasting plasma glucose (FPG) are listed. **Table S6**. Evaluation metrics for classification of type 2 diabetes patients and controls by combining the levels of 27 glycated peptides in tryptic plasma digests and corresponding HbA1c levels calculated by Decision Tree classifier from Scikit-learn package. **Table S7**. Evaluation metrics for classification of type 2 diabetes patients and controls by combining the levels of 27 glycated peptides in tryptic plasma digests and corresponding FPG levels. **Table S8**. Evaluation metrics for classification of type 2 diabetes patients and controls by combining the levels of 27 glycated peptides in tryptic plasma digests and corresponding HbA1c levels. Variable cut points were optimized manually for best classification. **Table S9**. Evaluation metrics for classification of type 2 diabetes patients and controls by combining the levels of 27 glycated peptides in tryptic plasma digests and corresponding fasting plasma glucose (FPG) levels. Variable cut points were optimized manually for best classification. **Figure S1**. Quantification of glycated peptides in tryptic plasma digests obtained from type 2 diabetes patients and non-diabetic controls using internal calibration. **Figure S2**. Dendrogram resulting from a hierarchical clustering of 48 type 2 diabetes patient samples considering all available patient information, i.e. diagnostic parameters and glycated peptide levels.

## Abbreviations

ADA: American Diabetes Association
AUC: area under the curve
BAC: boronic acid affinity chromatography
BMI: body mass index
CRP: C-reactive protein
CV: coefficient of variation
DM: diabetes mellitus
EM: expectation–maximation
ESI–MS: electrospray ionization mass spectrometry
FFA: free fatty acids
FPG: fasting plasma glucose
HbA_1c_: glycated hemoglobin
HDL: high-density lipoprotein
HOMA-IR: homeostasis model assessment as an index of insulin resistance
HP K141: glycated lysine-141 of haptoglobin
HSA: human serum albumin
LDL: low-density lipoprotein
LOD: limit of detection
LOQ: limit of quantification
MRM: multiple reaction monitoring
OGTT: oral glucose tolerance test
ROC: receiver operating characteristic
RP-HPLC: reversed-phase high-performance liquid chromatography
SPE: solid phase extraction
SVM-RFE: support vector machine-recursive feature elimination
T2DM: type 2 diabetes mellitus
WHO: World Health Organization
XIC: extracted ion chromatogram
