# Supplementary material for: Glycated lysine-141 in haptoglobin improves the diagnostic accuracy for type 2 diabetes mellitus in combination with glycated hemoglobin HbA1c and fasting plasma glucose
Source: Clin Proteomics. 2017 Mar 28;14:10. doi: 10.1186/s12014-017-9145-1 (PMC5370432; doi:10.1186/s12014-017-9145-1)
Supplement: Supplementary file 1 — Additional file 1. Table S1. Characterization of type 2 diabetes patients and matched non-diabetic persons enrolled in this study. Table S2. Parameters and settings used for the RP-HPLC-ESI-QqLIT-MS operating in scheduled multiple reaction monitoring (MRM) mode. Table S3. Precision, sensitivity and linearity parameters for glycated peptides. Table S4. Spearman rank correlation coefficients (rS) and corresponding P values (P) of the statistical relation between glycated peptides and several diagnostic parameters. Table S5. Receiver operating characteristic (ROC) parameters for peptide levels of all 27 glycated peptides quantified in tryptic digests of plasma samples obtained from 48 type 2 diabetes patients and 48 controls. For comparison, ROC parameters of HbA1C and fasting plasma glucose (FPG) are listed. Table S6. Evaluation metrics for classification of type 2 diabetes patients and controls by combining the levels of 27 glycated peptides in tryptic plasma digests and corresponding HbA1c levels calculated by Decision Tree classifier from Scikit-learn package. Table S7. Evaluation metrics for classification of type 2 diabetes patients and controls by combining the levels of 27 glycated peptides in tryptic plasma digests and corresponding FPG levels. Table S8. Evaluation metrics for classification of type 2 diabetes patients and controls by combining the levels of 27 glycated peptides in tryptic plasma digests and corresponding HbA1c levels. Variable cut points were optimized manually for best classification. Table S9. Evaluation metrics for classification of type 2 diabetes patients and controls by combining the levels of 27 glycated peptides in tryptic plasma digests and corresponding fasting plasma glucose (FPG) levels. Variable cut points were optimized manually for best classification. Figure S1. Quantification of glycated peptides in tryptic plasma digests obtained from type 2 diabetes patients and non-diabetic controls using internal calibration. Figure S2. D [file 12014_2017_9145_MOESM1_ESM.doc]

**Glycated lysine-141 in haptoglobin improves the diagnostic accuracy for type 2 diabetes mellitus in combination with glycated hemoglobin HbA_1c_ and fasting plasma glucose**

Sandro Spiller, Yichao Li, Matthias Blüher, Lonnie Welch, Ralf Hoffmann

**Electronic Supplementary Information**

Directory

Table S1 2

Table S2 3

Table S3 4

Table S4 5

Table S5 6

Table S6 7

Table S7 8

Table S8 9

Table S9 10

Figure S1 11

Figure S2 12

## **Table S1.** Characterization of type 2 diabetes patients and matched non-diabetic persons enrolled in this study.

| Variable | Control group | Diabetes patients  (HbA_1c_ < 6.5% (48 mmol/mol)) | Diabetes patients (HbA_1c_ ≥ 6.5%(48 mmol/mol)) |
| --- | --- | --- | --- |
| *n* | 48 | 23 | 25 |
| Age (years) | 56 ± 10 | 58 ± 8 | 56 ± 9 |
| Height (cm) | 178 ± 9 | 174 ± 7 | 177 ± 9 |
| Body weight (kg) | 106 ± 21 | 104 ± 16 | 107 ± 15 |
| BMI (kg/m^2^) | 33.5 ± 5.6 | 34.1 ± 4.2 | 34.1 ± 4.1 |
| HbA_1C_ (%) | 5.6 ± 0.3 | 5.8 ± 0.5 | 7.3 ± 0.8 |
| Fasting plasma glucose (mmol/L) | 5.2 ± 0.6 | 6.9 ± 2.0 | 7.1 ± 1.7 |
| Fasting plasma glucose (mg/dL) | 93.6 ± 10.8 | 124.2 ± 36.0 | 127.8 ± 30.6 |

Data are means ± SD for the subjects of each group.

## **Table S2**. Parameters and settings used for the RP-HPLC-ESI-QqLIT-MS operating in scheduled multiple reaction monitoring (MRM) mode.

| **Parameter** | **Settings** |
| --- | --- |
| IS [V] | 5500 |
| TEM [°C] | 650 |
| Nebulizer gas (Gas 1, psig) | 40 |
| Drying gas (Gas 2, psig) | 75 |
| Curtain gas (CUR, psig) | 35 |
| Declustering potential (DP, V) | - ^a^ |
| Collision cell entrance potential (EP, V) | 10 |
| Collision cell exit potential (CXP, V) | - ^a^ |
| Interface heating | on |
| Q_1_ resolution | unit |
| Q_3_ resolution | - |
| Q_3_ entry barrier (V) | - |
| Collision (CAD) gas | high |
| Collision potential (CE, V) | - ^a^ |
| *m/z* Range (Da) | - |
| Scan rate (Da/s) | - |
| Step size (Da) | - |
| Cycle time [s] | 1.35 |
| Dwell time [ms] | - ^b^ |

^a^ Parameters depended on peptide sequence and charge state (for specific precursor/fragment ion pair (Q1/Q3 mass range) see Table 1), ^b^ automatically defined by Scheduled MRM™ Algorithm.

## **Table S3.** Precision, sensitivity and linearity parameters for glycated peptides.

| **#***** | **LOD**  **[nmol/L]** | **LOQ**  **[nmol/L]** | **LDR** | **Intraday precision (n = 3)**  **Content [pmol/mg] ± SD**  **(RSD%)** | **Interday precision (n = 3/day)**  **Content [pmol/mg] ± SD**  **(RSD%)** |
| --- | --- | --- | --- | --- | --- |
| **1** | 2.0 | 2.0 | 1000 | 30.0 ± 0.3 (1) | 31.8 ± 2.5 (8) |
| **2** | 5.0 | 5.0 | 200 | 7.7 ± 0.0 (0) | 7.5 ± 0.5 (7) |
| **3** | 2.0 | 5.0 | 200 | 3.5 ± 0.1 (3) | 3.1 ± 0.3 (10) |
| **4** | 2.0 | 2.0 | 500 | 11.3 ± 1.5 (13) | 10.1 ± 2.0 (20) |
| **5** | 5.0 | 10.0 | 500 | 75.7 ± 1.9 (3) | 74.0 ± 7.0 (10) |
| **6** | 5.0 | 10.0 | 200 | 123.6 ± 2.8 (2) | 126.3 ± 10.7 (8) |
| **7** | 50.0 | 100.0 | 20 | 374.4 ± 13.0 (4) | 351.3 ± 41.2 (12) |
| **8** | 2.0 | 5.0 | 400 | 20.2 ± 0.4 (2) | 22.2 ± 2.4 (12) |
| **9** | 5.0 | 10.0 | 100 | 77.6 ± 3.7 (5) | 78.1 ± 6.4 (8) |
| **10** | 50.0 | 100.0 | 20 | 439.3 ± 11.1 (3) | 406.6 ± 88.1 (22) |
| **11** | 2.0 | 5.0 | 100 | 1.9 ± 0.0 (2) | 1.9 ± 0.1 (7) |
| **12** | 2.0 | 2.0 | 1000 | 2194.9± 31.4 (4) | 2191.7 ± 117.9 (5) |
| **13** | 100.0 | 200.0 | 25 | 31.4 ± 1.0 (3) | 34.5 ± 7.9 (23) |
| **14** | 2.0 | 5.0 | 200 | 19.6 ± 0.4 (2) | 18.0 ± 1.9 (11) |
| **15** | 2.0 | 5.0 | 400 | 3.1 ± 0.2 (6) | 3.3 ± 0.5 (14) |
| **16** | 20.0 | 50.0 | 40 | 78.7 ± 0.5 (1) | 79.4 ± 1.4 (2) |
| **17** | 2.0 | 5.0 | 200 | 1.9 ± 0.0 (1) | 1.9 ± 0.1 (7) |
| **18** | 5.0 | 10.0 | 100 | 2.0 ± 0.0 (1) | 1.9 ± 0.2 (9) |
| **19** | 2.0 | 2.0 | 1000 | 0.3 ± 0.0 (3) | 0.3 ± 0.1 (20) |
| **20** | 20.0 | 50.0 | 400 | 19.6 ± 1.2 (6) | 19.1 ± 2.6 (14) |
| **21** | 2.0 | 5.0 | 200 | 1.4 ± 0.0 (3) | 1.6 ± 0.3 (21) |
| **22** | 2.0 | 5.0 | 400 | 1.5 ± 0.0 (0) | 1.5 ± 0.2 (10) |
| **23** | 2.0 | 2.0 | 500 | 0.9 ± 0.0 (3) | 0.9 ± 0.1 (9) |
| **24** | 2.0 | 5.0 | 400 | 1.6 ± 0.1 (4) | 1.8 ± 0.3 (15) |
| **25** | 2.0 | 2.0 | 1000 | 0.037 ± 0.001 (4) | 0.037 ± 0.005 (12) |
| **26** | 2.0 | 2.0 | 500 | 0.6 ± 0.1 (14) | 0.6 ± 0.2 (30) |
| **27** | 5.0 | 5.0 | 200 | 0.8 ± 0.0 (2) | 0.8 ± 0.1 (14) |

## ^*^Sequence numbers corresponds to peptides listed in Table 1. LOD - limit of detection, LOQ - limit of quantification, LDR - linear dynamic range. Sensitivity and linearity parameters were obtained by external calibration injecting three independent dilution series (10 µmol/L to 10 pmol/L) of authentic synthetic peptide mixtures. Intra- and interday precision values were determined in pooled plasma (48 diabetic and 48 control samples) using the stable isotope dilution approach (SIDA) and external calibration. Interday precision was performed on three consecutive days.

## **Table S4.** Spearman rank correlation coefficients (*r_S_*) and corresponding P-values (*P*) of the statistical relation between glycated peptides and several diagnostic parameters.

| **#***** | **BMI**  **[kg/m^2^]** | | **Body fat**  **[%]** | | **C-peptide**  **[nmol/L]** | | **HbA_1c_**  **[%]** | | **FPG**  **[mmol/L]** | |
| --- | --- | --- | --- | --- | --- | --- | --- | --- | --- | --- |
|  | *r_S_* | *P* | *r_S_* | *P* | *r_S_* | *P* | *r_S_* | *P* | *r_S_* | *P* |
| **1** | -0.38 | 1.6E-04 | -0.27 | 2.7E-02 | -0.39 | 8.9E-04 | 0.42 | 2.2E-05 | 0.18 | 7.9E-02 |
| **2** | -0.50 | 2.6E-07 | -0.36 | 2.5E-03 | -0.44 | 1.9E-04 | 0.31 | 2.0E-03 | 0.17 | 9.6E-02 |
| **3** | -0.54 | 1.8E-08 | -0.48 | 4.7E-05 | -0.33 | 4.9E-03 | 0.14 | 1.6E-01 | 0.09 | 4.0E-01 |
| **4** | -0.51 | 1.0E-07 | -0.49 | 2.1E-05 | -0.35 | 2.8E-03 | 0.11 | 2.9E-01 | 0.10 | 3.2E-01 |
| **5** | -0.51 | 9.5E-08 | -0.41 | 5.3E-04 | -0.43 | 2.1E-04 | 0.26 | 1.1E-02 | 0.16 | 1.1E-01 |
| **6** | -0.35 | 4.9E-04 | -0.27 | 2.5E-02 | -0.40 | 6.8E-04 | 0.34 | 6.4E-04 | 0.19 | 6.0E-02 |
| **7** | -0.37 | 1.7E-04 | -0.22 | 6.8E-02 | -0.29 | 1.6E-02 | 0.43 | 1.0E-05 | 0.12 | 2.3E-01 |
| **8** | -0.32 | 1.4E-03 | -0.26 | 3.1E-02 | -0.45 | 1.2E-04 | 0.39 | 9.0E-05 | 0.26 | 1.1E-02 |
| **9** | -0.51 | 1.0E-07 | -0.38 | 1.3E-03 | -0.45 | 1.2E-04 | 0.27 | 7.5E-03 | 0.20 | 4.6E-02 |
| **10** | -0.48 | 6.6E-07 | -0.37 | 2.3E-03 | -0.40 | 6.4E-04 | 0.32 | 1.3E-03 | 0.12 | 2.6E-01 |
| **11** | -0.45 | 3.3E-06 | -0.34 | 5.6E-03 | -0.37 | 1.6E-03 | 0.33 | 1.2E-03 | 0.20 | 4.9E-02 |
| **12** | -0.40 | 6.2E-05 | -0.32 | 8.5E-03 | -0.32 | 7.0E-03 | 0.37 | 1.8E-04 | 0.14 | 1.6E-01 |
| **13** | -0.34 | 6.6E-04 | -0.24 | 5.2E-02 | -0.31 | 8.7E-03 | 0.45 | 3.5E-06 | 0.21 | 3.9E-02 |
| **14** | -0.45 | 3.4E-06 | -0.38 | 1.3E-03 | -0.38 | 1.2E-03 | 0.34 | 5.9E-04 | 0.18 | 8.3E-02 |
| **15** | -0.10 | 3.1E-01 | -0.04 | 7.4E-01 | -0.24 | 4.3E-02 | 0.39 | 7.5E-05 | 0.19 | 6.5E-02 |
| **16** | -0.13 | 2.0E-01 | 0.02 | 8.6E-01 | -0.23 | 5.7E-02 | 0.43 | 1.4E-05 | 0.24 | 1.7E-02 |
| **17** | -0.12 | 2.4E-01 | -0.09 | 4.6E-01 | -0.26 | 3.1E-02 | 0.30 | 2.6E-03 | 0.19 | 6.5E-02 |
| **18** | -0.25 | 1.3E-02 | -0.28 | 2.1E-02 | -0.33 | 5.6E-03 | 0.38 | 1.5E-04 | 0.20 | 4.9E-02 |
| **19** | -0.28 | 5.0E-03 | -0.38 | 1.4E-03 | -0.31 | 8.9E-03 | 0.30 | 3.4E-03 | 0.23 | 2.5E-02 |
| **20** | 0.16 | 1.3E-01 | -0.09 | 4.5E-01 | -0.14 | 2.4E-01 | 0.19 | 6.6E-02 | 0.32 | 1.4E-03 |
| **21** | -0.51 | 1.3E-07 | -0.44 | 2.0E-04 | -0.31 | 1.0E-02 | 0.20 | 5.6E-02 | 0.10 | 3.4E-01 |
| **22** | -0.32 | 1.6E-03 | -0.32 | 8.6E-03 | -0.29 | 1.7E-02 | 0.28 | 5.9E-03 | 0.30 | 2.6E-03 |
| **23** | -0.54 | 1.6E-08 | -0.39 | 1.2E-03 | -0.30 | 1.1E-02 | 0.14 | 1.6E-01 | 0.04 | 6.7E-01 |
| **24** | -0.53 | 2.6E-08 | -0.47 | 6.3E-05 | -0.32 | 7.8E-03 | 0.14 | 1.8E-01 | 0.10 | 3.4E-01 |
| **25** | -0.35 | 4.2E-04 | -0.39 | 9.7E-04 | -0.26 | 2.9E-02 | 0.20 | 5.1E-02 | 0.21 | 3.5E-02 |
| **26** | -0.35 | 5.6E-04 | -0.27 | 2.7E-02 | -0.28 | 2.0E-02 | 0.18 | 8.4E-02 | -0.08 | 4.2E-01 |
| **27** | -0.39 | 8.6E-05 | -0.28 | 2.2E-02 | -0.17 | 1.6E-01 | 0.17 | 9.6E-02 | 0.05 | 6.4E-01 |

## ^*^Sequence numbers corresponds to peptides listed in Table 1. BMI - body mass index, HbA_1c_ - glycated hemoglobin, FPG - fasting plasma glucose.

**Table S5:** Receiver operating characteristic (ROC) analysis. ROC parameters were estimated for peptide levels of all 27 glycated peptides quantified in tryptic digests of plasma samples obtained from 48 type 2 diabetes patients and 48 controls. For comparison, ROC parameters of HbA_1C_ and fasting plasma glucose (FPG) are listed.

| **Tested**  **variable** | **AUC**^†^  **(%)** | **Sensitivity**  **(%)** | **Specificity**  **(%)** | **Cut-off** |
| --- | --- | --- | --- | --- |
| **Sequence #*^*^*** |  |  |  | **(pmol/mg plasma protein)** |
| **1** | 76 | 77 | 63 | 27.5 |
| **2** | 75 | 79 | 56 | 4.3 |
| **3** | 68 | 56 | 81 | 1.9 |
| **4** | 69 | 62 | 71 | 4.8 |
| **5** | 75 | 54 | 85 | 55.0 |
| **6** | 73 | 73 | 63 | 110.0 |
| **7** | 69 | 69 | 67 | 177.0 |
| **8** | 79 | 69 | 71 | 23.1 |
| **9** | 76 | 58 | 83 | 50.6 |
| **10** | 74 | 58 | 79 | 57.5 |
| **11** | 79 | 60 | 88 | 1.27 |
| **12** | 71 | 77 | 60 | 1,930.0 |
| **13** | 74 | 79 | 63 | 121.0 |
| **14** | 75 | 56 | 83 | 14.7 |
| **15** | 67 | 69 | 62 | 4.77 |
| **16** | 68 | 69 | 65 | 35.9 |
| **17** | 66 | 50 | 83 | 2.3 |
| **18** | 77 | 69 | 81 | 1.3 |
| **19** | 74 | 69 | 69 | 0.5 |
| **20** | 68 | 56 | 75 | 14.1 |
| **21** | 70 | 56 | 73 | 3.0 |
| **22** | 75 | 62 | 83 | 1.1 |
| **23** | 67 | 67 | 65 | 0.9 |
| **24** | 69 | 60 | 75 | 1.3 |
| **25** | 77 | 60 | 83 | 0.02 |
| **26** | 62 | 67 | 52 | 0.8 |
| **27** | 65 | 67 | 60 | 0.9 |
|  |  |  |  | **(%)** |
| **HbA_1c_** | 86 | 77 | 94 | 6.0 |
|  | 86 | 52 | 100 | 6.5 |
|  |  |  |  | **(mmol/L)** |
| **FPG** | 84 | 75 | 81 | 5.69 |
|  | 84 | 40 | 100 | 7.00 |

^*^Sequence numbers corresponds to peptides listed in Table 1. ^†^AUC - Area under the curve.

**Table S6.** Evaluation metrics for classification of type 2 diabetes patients and controls by combining the levels of 27 glycated peptides in tryptic plasma digests and corresponding HbA_1c_ levels calculated by Decision Tree classifier from scikit-learn package. The optimal cut point for HbA_1c_ was 6.05% (43 mmol/mol) for all combinations.

| **Sequence #*^*^*** | **Sensitivity (%)** | **Specificity (%)** | **Accuracy (%)** | **Cut-off**^†^  **(pmol/mg plasma protein)** |
| --- | --- | --- | --- | --- |
| **1** | 75 | 100 | 88 | 36.56 |
| **2** | 75 | 100 | 88 | 7.09 |
| **3** | 75 | 100 | 88 | - |
| **4** | 75 | 100 | 88 | - |
| **5** | 75 | 100 | 88 | 64.85 |
| **6** | 75 | 100 | 88 | - |
| **7** | 75 | 100 | 88 | - |
| **8** | 75 | 100 | 88 | 35.11 |
| **9** | 75 | 100 | 88 | - |
| **10** | 75 | 100 | 88 | 71.96 |
| **11** | 75 | 100 | 88 | 1.48 |
| **12** | 75 | 100 | 88 | - |
| **13** | 75 | 100 | 88 | - |
| **14** | 75 | 100 | 88 | - |
| **15** | 75 | 100 | 88 | - |
| **16** | 75 | 100 | 88 | - |
| **17** | 75 | 98 | 87 | 2.69 |
| **18** | 77 | 96 | 87 | - |
| **19** | 75 | 100 | 88 | - |
| **20** | 75 | 100 | 88 | - |
| **21** | 75 | 100 | 88 | - |
| **22** | 81 | 98 | 90 | 1.36 |
| **23** | 75 | 98 | 87 | - |
| **24** | 79 | 100 | 90 | 1.94 |
| **25** | 94 | 98 | 96 | 0.03 |
| **26** | 75 | 100 | 88 | - |
| **27** | 75 | 100 | 88 | - |

^*^Sequence numbers correspond to peptides listed in Table 1. ^†^Missing value means that the decision tree algorithm did not choose the feature. The reason for not giving a cut-off value is because the addition of the feature will decrease the classification accuracy.

**Table S7.** Evaluation metrics for classification of type 2 diabetes patients and controls by combining the levels of 27 glycated peptides in tryptic plasma digests and corresponding FPG levels calculated by Decision Tree classifier from scikit-learn package. The optimal cut point for FPG was 5.98 mmol/L for all combinations.

| **Sequence #*^*^*** | **Sensitivity (%)** | **Specificity (%)** | **Accuracy (%)** | **Cut-off**^†^  **(pmol/mg plasma protein)** |
| --- | --- | --- | --- | --- |
| **1** | 76 | 96 | 86 | 35.11 |
| **2** | 72 | 98 | 85 | 7.03 |
| **3** | 70 | 92 | 81 | - |
| **4** | 68 | 92 | 80 | - |
| **5** | 68 | 96 | 82 | 74.03 |
| **6** | 66 | 94 | 80 | - |
| **7** | 76 | 98 | 87 | 235.01 |
| **8** | 84 | 94 | 89 | 28.44 |
| **9** | 74 | 98 | 86 | 6.66 |
| **10** | 76 | 98 | 87 | 72.73 |
| **11** | 78 | 96 | 87 | 1.53 |
| **12** | 74 | 98 | 86 | 2583.69 |
| **13** | 68 | 98 | 83 | 157.93 |
| **14** | 76 | 100 | 88 | 18.98 |
| **15** | 66 | 96 | 81 | - |
| **16** | 66 | 98 | 82 | - |
| **17** | 70 | 96 | 83 | 2.72 |
| **18** | 76 | 94 | 85 | 1.71 |
| **19** | 64 | 100 | 82 | - |
| **20** | 64 | 100 | 82 | - |
| **21** | 68 | 96 | 82 | 3.59 |
| **22** | 74 | 98 | 86 | 1.33 |
| **23** | 66 | 92 | 79 | - |
| **24** | 66 | 100 | 83 | 1.84 |
| **25** | 78 | 98 | 88 | 0.04 |
| **26** | 66 | 100 | 83 | 0.88 |
| **27** | 66 | 92 | 79 | - |

^*^Sequence numbers correspond to peptides listed in Table 1. ^†^Missing value means that the decision tree algorithm did not choose the feature. The reason for not giving a cut-off value is because the addition of the feature will decrease the classification accuracy.

## **Table S8.** Evaluation metrics for classification of type 2 diabetes patients and controls by combining the levels of 27 glycated peptides in tryptic plasma digests and corresponding HbA_1c_ levels. Variable cut points were optimized manually for best classification. The optimal cut point for HbA_1c_ was 6.0% (42 mmol/mol) for all combinations.

| **Sequence #*^*^*** | **Sensitivity (%)** | **Specificity (%)** | **Accuracy (%)** | **Cut-off**  **(pmol/mg plasma protein)** |
| --- | --- | --- | --- | --- |
| **1** | 79 | 100 | 90 | 35.50 |
| **2** | 81 | 100 | 91 | 7.00 |
| **3** | 81 | 98 | 90 | 5.10 |
| **4** | 85 | 98 | 92 | 2.10 |
| **5** | 85 | 98 | 92 | 65.00 |
| **6** | 81 | 96 | 89 | 14.40 |
| **7** | 75 | 100 | 88 | 231.00 |
| **8** | 83 | 96 | 90 | 28.00 |
| **9** | 83 | 98 | 91 | 6.00 |
| **10** | 81 | 98 | 90 | 64.00 |
| **11** | 85 | 100 | 93 | 1.47 |
| **12** | 77 | 98 | 88 | 2530.00 |
| **13** | 79 | 98 | 89 | 155.00 |
| **14** | 83 | 94 | 89 | 16.10 |
| **15** | 81 | 90 | 85 | 6.00 |
| **16** | 81 | 90 | 85 | 39.50 |
| **17** | 83 | 98 | 91 | 2.50 |
| **18** | 90 | 94 | 92 | 1.62 |
| **19** | 79 | 100 | 90 | 0.70 |
| **20** | 81 | 90 | 85 | 16.90 |
| **21** | 81 | 98 | 90 | 4.00 |
| **22** | 88 | 98 | 93 | 1.32 |
| **23** | 83 | 98 | 91 | 1.27 |
| **24** | 88 | 96 | 92 | 1.86 |
| **25** | 94 | 98 | 96 | 0.03 |
| **26** | 79 | 96 | 88 | 0.84 |
| **27** | 81 | 94 | 88 | 1.05 |

^*^Sequence numbers correspond to peptides listed in Table 1.

**Table S9.** Evaluation metrics for classification of type 2 diabetes patients and controls by combining the levels of 27 glycated peptides in tryptic plasma digests and corresponding fasting plasma glucose (FPG) levels. Variable cut points were optimized manually for best classification. The optimal cut point for FPG was 6.0 mmol/L for all combinations.

| **Sequence #*^*^*** | **Sensitivity (%)** | **Specificity (%)** | **Accuracy (%)** | **Cut-off**  **(pmol/mg plasma protein)** |
| --- | --- | --- | --- | --- |
| **1** | 81 | 98 | 90 | 35.50 |
| **2** | 79 | 98 | 89 | 7.00 |
| **3** | 79 | 94 | 86 | 5.10 |
| **4** | 81 | 92 | 86 | 2.10 |
| **5** | 81 | 90 | 85 | 65.00 |
| **6** | 81 | 92 | 86 | 14.40 |
| **7** | 79 | 98 | 89 | 231.00 |
| **8** | 85 | 96 | 91 | 28.00 |
| **9** | 85 | 85 | 85 | 6.00 |
| **10** | 79 | 98 | 89 | 64.00 |
| **11** | 85 | 94 | 90 | 1.47 |
| **12** | 77 | 85 | 81 | 2530.00 |
| **13** | 79 | 98 | 89 | 155.00 |
| **14** | 81 | 94 | 88 | 16.10 |
| **15** | 75 | 96 | 85 | 6.00 |
| **16** | 77 | 90 | 83 | 39.50 |
| **17** | 81 | 92 | 86 | 2.50 |
| **18** | 88 | 94 | 91 | 1.62 |
| **19** | 75 | 96 | 85 | 0.70 |
| **20** | 75 | 96 | 85 | 16.90 |
| **21** | 81 | 96 | 89 | 4.00 |
| **22** | 79 | 98 | 89 | 1.32 |
| **23** | 83 | 88 | 85 | 1.27 |
| **24** | 81 | 90 | 85 | 1.86 |
| **25** | 83 | 98 | 91 | 0.03 |
| **26** | 75 | 92 | 83 | 0.84 |
| **27** | 81 | 94 | 88 | 1.05 |

^*^Sequence numbers corresponds to peptides listed in Table 1.

##


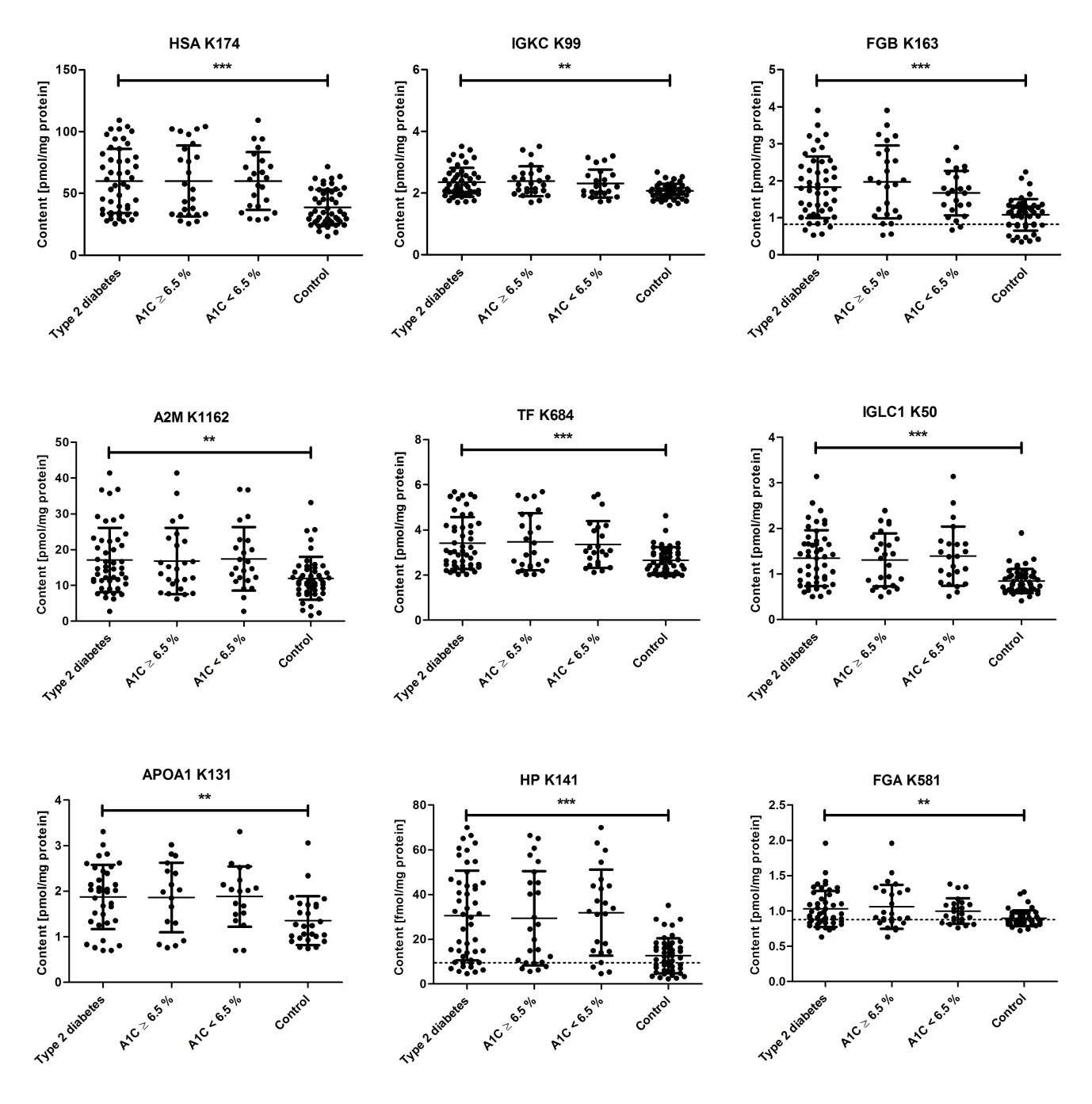


## **Figure S1.** Quantification of glycated peptides in tryptic plasma digests obtained from type 2 diabetes patients and non-diabetic controls using internal calibration. Peptide codes above each graph combine the protein code and glycation site (Table 1). Additionally, samples were split in two groups using an HbA_1C_ level of 6.5 % (48 mmol/mol) as cut-off. Each dot represents the peptide level of the corresponding peptide in one plasma sample. Dotted lines indicate the lower limits of quantification (LLOQ) of the corresponding peptide. Statistical significance was tested by a Mann-Whitney U-test (*** denotes *p* < 0.0001 and ** denotes *p* < 0.01).


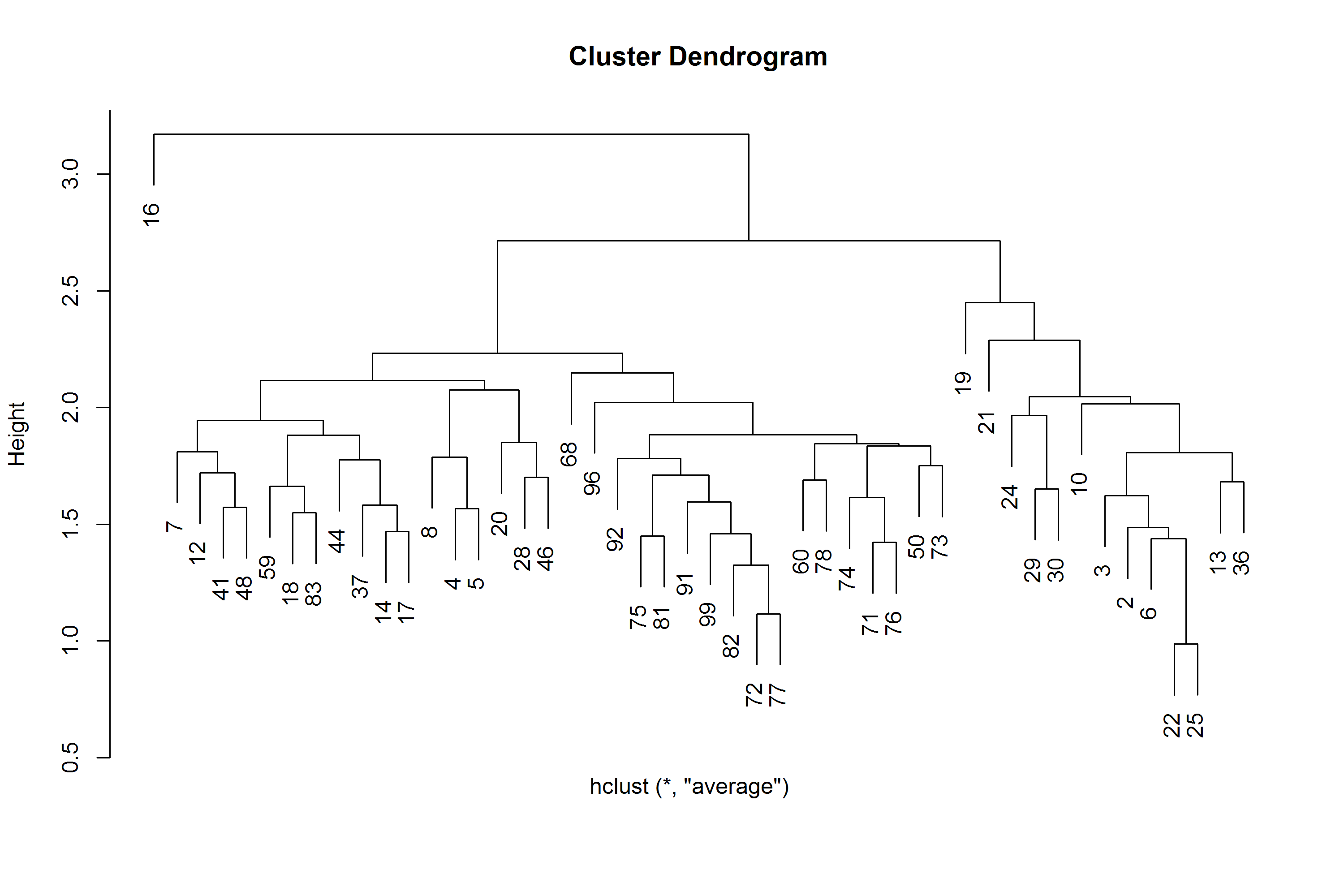


## **Figure S2.** Dendrogram resulting from a hierarchical clustering of 48 type 2 diabetes patient samples considering all available patient information, i.e. diagnostic parameters and glycated peptide levels. Missing values were imputed using Weka.
